# Acute Effects of Aerosolized Iloprost in COPD Related Pulmonary Hypertension - A Randomized Controlled Crossover Trial

**DOI:** 10.1371/journal.pone.0052248

**Published:** 2012-12-27

**Authors:** Lucas Boeck, Michael Tamm, Peter Grendelmeier, Daiana Stolz

**Affiliations:** Clinic of Pulmonary Medicine and Respiratory Cell Research, University Hospital Basel, Basel, Switzerland; The Ohio State Unversity, United States of America

## Abstract

**Background:**

Inhaled iloprost potentially improves hemodynamics and gas exchange in patients with chronic obstructive pulmonary disease (COPD) and secondary pulmonary hypertension (PH).

**Objectives:**

To evaluate acute effects of aerosolized iloprost in patients with COPD-associated PH.

**Methods:**

A randomized, double blind, crossover study was conducted in 16 COPD patients with invasively confirmed PH in a single tertiary care center. Each patient received a single dose of 10 µg iloprost (low dose), 20 µg iloprost (high dose) and placebo during distinct study-visits. The primary end-point of the study was exercise capacity as assessed by the six minute walking distance.

**Results:**

Both iloprost doses failed to improve six-minute walking distance (p = 0.36). Low dose iloprost (estimated difference of the means −1.0%, p = 0.035) as well as high dose iloprost (−2.2%, p<0.001) significantly impaired oxygenation at rest. Peak oxygen consumption and carbon dioxide production differed significantly over the three study days (p = 0.002 and p = 0.003, accordingly). As compared to placebo, low dose iloprost was associated with reduced peak oxygen consumption (−76 ml/min, p = 0.002), elevated partial pressure of carbon dioxide (0.27 kPa, p = 0.040) and impaired ventilation during exercise (−3.0l/min, p<0.001).

**Conclusions:**

Improvement of the exercise capacity after iloprost inhalation in patients with COPD-associated mild to moderate PH is very unlikely.

**Trial Registration:**

Controlled-Trials.com ISRCTN61661881

## Introduction

Chronic obstructive pulmonary disease (COPD) is a leading cause of morbidity and mortality worldwide [1]. The increasing prevalence of COPD demands substantial progress to prevent and control the enormous burden of disease. COPD patients with secondary pulmonary hypertension (PH) have more severe disease, more frequent exacerbations, more rapid decline of functional capacity and worse outcome [2], [3], [4]. Thus, cardiovascular disease mechanisms are gaining importance as potential treatment targets in COPD [5], [6], [7].

So far, only a few trials investigated pulmonary vasodilators in COPD patients [8], [9], [10], [11]. Unfortunately, pulmonary vasodilators mostly caused an impaired gas exchange and did not improve exercise capacity in COPD. Hereby it is conceivable that non-selective (ventilation-independent) pulmonary vasodilatation and inhibition of hypoxic vasoconstriction increases ventilation/perfusion (V/Q) mismatch and intrapulmonary shunting. Drug application by inhalation might overcome this flaw. Inhaled medications facilitate access to alveolar units, which receive the greatest proportion of ventilation, hence redirecting pulmonary blood flow advantageously to these areas while lowering pulmonary pressures. Two inhaled vasodilators, namely nitric oxide and prostacyclin, have shown to improve oxygenation and pulmonary artery pressure (PAP) in high V/Q mismatch states [12], [13]. Although nitric oxide has shown beneficial aspects in COPD, its use is inconvenient and the safety of this approach has been considered questionable [14], [15], [16]. In contrast, prostacyclin, one of the most powerful pulmonary vasodilators, might play a central role in the development of secondary PH in COPD [17], [18]. The selective vasodilative properties of inhaled iloprost, a prostacyclin analogue, were previously investigated in interstitial lung disease [19]. Recently, an open-label study reported an improvement in 6 minute walking distance after iloprost inhalation in COPD patients [20]. Importantly, V/Q matching improved after iloprost administration. However, to our knowledge, inhaled prostacyclins have not been investigated in a randomized trial in COPD patients.

The aim of this study was to evaluate whether inhaled iloprost improves exercise capacity in COPD patients with pulmonary hypertension in a randomized, double-blind fashion.

## Methods

The study was designed as a prospective, randomized, double-blind, single center, cross-over trial comparing two doses of inhaled iloprost (10 µg iloprost = low dose iloprost and 20 µg iloprost = high dose iloprost) and placebo in 16 patients with COPD-associated PH (a detailed description of the methods is provided by the supporting information – Supporting information S1, Protocol S1, CONSORT checklist S1). The trial was conducted from October 2009 to September 2010 at the University Hospital Basel. The study protocol was reviewed and approved by the Institutional Review Board (EKBB 190/2009) and registered as the OPTION trial (On demand prostacyclin inhalation in obstructive pulmonary disease and pulmonary hypertension; ISRCTN61661881). Written informed consent was obtained from all participants before study inclusion.

### Patients

**Figure 1:**
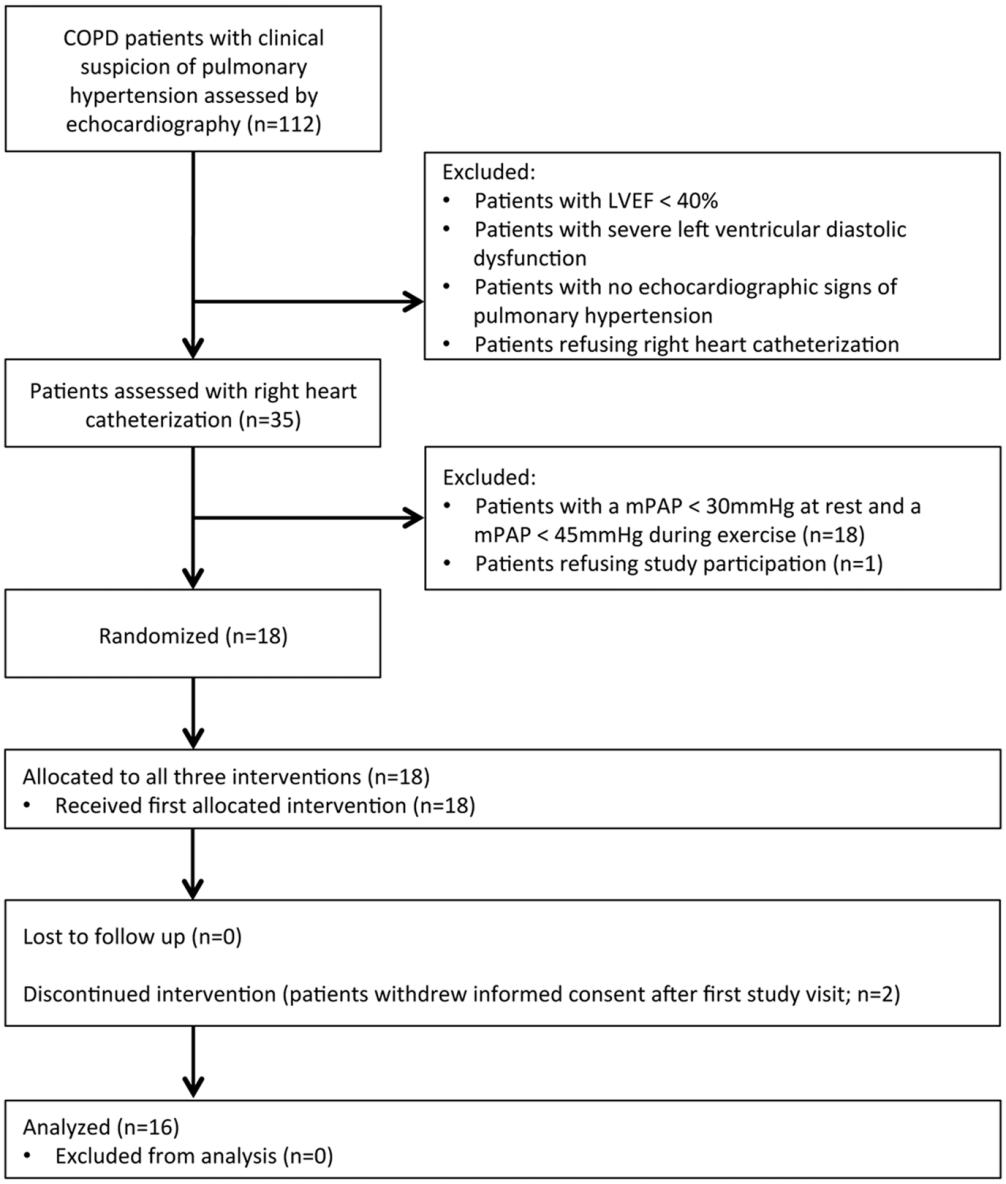
Screening, enrollment and interventions of the study participants. COPD denotes chronic obstructive pulmonary disease, CVP central venous pressure, LVEF left ventricular ejection fraction, mPAP mean pulmonary artery pressure, PH pulmonary hypertension, sPAP systolic pulmonary artery pressure, 6MWT six-minute walking distance.

Patients with a smoking history of more than 20 pack-years and confirmed COPD, as specified by the global initiative of chronic obstructive lung disease (GOLD) guidelines [21], were considered for eligibility according to a screening algorithm (**Figure 1**). Patients were included if 1) COPD was confirmed by lung function, 2) age was above 40 years, 3) the mean pulmonary artery pressure (mPAP) at rest was ≥ 30 mmHg and/or mPAP during exercise was ≥ 45 mmHg, 4) COPD management adhered to GOLD guidelines. Patients were excluded if 1) severe mental disorder prevented appropriate judgment concerning study participation, 2) life expectancy was severely restricted (less than 6 months), 3) COPD exacerbated within the last 4 weeks or changes in COPD management occurred within less than 3 months, 4) there were significant signs of left heart failure, 5) the patient had a history of pulmonary embolism, 6) PH was explained by another cause than COPD, including hypoventilation syndrome and/or sleep apnea syndrome, 7) the patient was pregnant or breastfeeding. In patients with insufficient COPD management, therapy was optimized according to GOLD guidelines and study inclusion postponed by 3 months. One patient declining right heart catheterization with estimated systolic pulmonary artery pressure on echocardiography of 65 mmHg in addition to the central venous pressure was included in the study.

### Study Procedures

Formulas for inhalation were randomized by an automated computer-generated randomization scheme and assigned to specific study days. The patient as well as the study personnel, who administered the inhalation and performed all tests, was blinded to the medication allocation. A nurse and a physician, responsible for preparation of the medication, were the only persons aware of the randomization code during the trial. They were not involved in other study functions. Every patient received 10 µg iloprost (low dose iloprost), 20 µg iloprost (high dose iloprost) or placebo (normal saline) on three different study days. Iloprost (Ventavis®) was diluted in normal saline to achieve a 2 ml solution. The three solutions were not visually distinguishable. Approximately 15 minutes before inhalation of the study medication a short acting beta-2 agonist (200 µg salbutamol; Ventolin®) was inhaled via a spacer. Placebo and iloprost was inhaled through an ultrasonic nebulizer system (Multisonic® infracontrol; Schill, Probstzella, Germany) [22].

#### Pulmonary function testing

Dynamic and static pulmonary function parameters were evaluated by body plethysmography. Carbon monoxide diffusing capacity (DLCO) was measured. All tests were performed according to the European Respiratory Society standards [23].

#### Echocardiography

Transthoracic two-dimensional and Doppler echocardiography were carried out by an ultrasound instrument (Philips, iE33) and performed in all patients for screening. Two-dimensional and Doppler imaging was performed in standard parasternal and apical views. Systolic pulmonary artery pressures (sPAP) were estimated from the systolic transtricuspid pressure gradient by means of the modified Bernoulli equation (tricuspid pressure gradient = 4×[maximal velocity of tricuspid regurgitant jet]^2^ ). Tricuspid annular plane systolic excursion (TAPSE) was estimated by two-dimensional echo guided M-mode recordings from the apical four-chamber view. According to the ESC/ERS guidelines, patients with possible or likely echocardiographic PH criteria, preserved systolic and absence of significant diastolic dysfunction were referred for right heart catheterization [24].

#### Right heart catheterization

Standard right heart catheterization measurements were taken without sedation from the right atrium, right ventricle and pulmonary artery at the end of expiration. Cardiac output was determined by the thermodilution method, whereas cardiac index is corrected for body surface area. Patients with normal or slightly elevated pulmonary pressures at rest were further evaluated during exercise.

#### Mobile cardiopulmonary exercise testing

The mobile cardiopulmonary exercise test was performed exactly 10 minutes after iloprost or placebo inhalation as described previously [25], [26]. Exercise parameters were measured using a telemetric mobile cardiopulmonary exercise test device (Oxycon Mobile® software v. 4.6, VIASYS Healthcare GmbH, Würzburg, Germany). This device consists of an EKG-triggered belt, an oxygen sensor, a facemask with a dead space <30 ml, a flow sensor, a sensor unit to measure oxygen and carbon dioxide, a data storage unit and a data transfer unit with integrated long-range telemetry, allowing real-time monitoring of the data. Heart rate, oxygen saturation, respiratory rate, tidal volume, oxygen consumption (VO2) and carbon dioxide production (VCO2) were continuously registered. Out of those parameters ventilation (VE), ventilatory reserve, O2 pulse, and ventilatory equivalents (VE/VO2, VE/CO2) were calculated. Before the exercise test there was a resting phase of several minutes. Measures at rest were obtained during a steady state after inhalation before exercise start. 10 minutes after inhalation of iloprost/placebo a six-minute walking test (6MWT) with the mobile exercise equipment was performed according to the American Thoracic Society guidelines [27]. At all times patients had the opportunity to slow the pace, to stop temporarily and to discontinue the test. During the test patients were not encouraged to walk faster or to continue walking. Exercise measures were obtained at peak oxygen uptake. Except for oxygen saturation, where minimal values, and heart rate where maximal values during exercise were analyzed. After test measures were acquired 6 minutes after exercise stop. Walking distance, perceived dyspnea as well as complications/side effects were recorded after the tests.

#### Six-Minute walk test

The 6MWT without cardiopulmonary exercise equipment was performed in patients who did not tolerate the facemask and/or strongly required oxygen during exercise. Inhalation, resting and the 6MWT was carried out identically. Heart rate and oxygen saturation were monitored continuously throughout the test. Walking distance, perceived dyspnea and complications/side effects were recorded after the test. 6MWTs were performed with a steady amount of oxygen at each of the three tests.

#### Arterial blood gas analysis

Immediately after each exercise test a standard arterial puncture to obtain a specimen for blood gas analysis was performed.

### Power Calculation

Power was calculated using the 6MWT distance before and after treatment as the primary outcome variable. Assuming a standard deviation of the difference before and after treatment of 50 m, there is a power >80% to detect a mean difference of 40 m with a sample size of 16 subjects by a two sided paired t-test (alpha = 0.05).

### Data Analysis

Discrete variables are expressed as counts (percentages) and continuous variables as mean ± standard deviation (SD). Parameters were analyzed by mixed-effects models, including treatment and period as fixed effects and subject as random effect. Results are presented as differences of means between placebo and iloprost with corresponding 95% confidence intervals and p-values. All tests were two tailed; p<0.05 was defined as significant. Data were analyzed using statistical software (Statistical Package for Social Sciences, IBM SPSS statistics 19, Chicago IL; R Development Core Team, version 2.9.2, Vienna).

## Results

Sixteen patients (10 men, 6 women) were included in the study (**Figure 1**). All patients had confirmed COPD and had a mean of 50 pack years ±29 smoked (mean ± standard deviation). Most patients had severe (25%) or very severe COPD (38%). Signs of emphysema were evident in 87%, air trapping in 100% and hyperinflation in 38%. At rest, 5 patients (31%) were hypoxemic and 5 patients (31%) hypercapnic. Demographic, pulmonary and hemodynamic characteristics of the study population are presented in **Table 1**. Included patients had a considerable number of comorbidities (**Table 2**). All patients received, at least, agents recommended by the global initiative of obstructive lung disease. 6 patients (38%) received nocturnal or long-term oxygen therapy.

**Table 1:** Population characteristics; discrete variables are expressed as counts (%) and continuous variables as mean ± standard deviation.

N	16
Age, years	73.2±6.7
Male gender (%)	10 (62.5%)
BMI	26.7±4.3
Smoked pack years	50±29
Exacerbation within the last year	1.2±1.4
MMRC	3.8±1.1
FEV_1_, liters	1.1±0.5
FEV_1_, % predicted	51.3±31.4
FEV_1_/FVC	44.2±16.8
TLC, liters	6.4±2.3
TLC, % predicted	107.2±29.7
RV/TLC	55.3±9.2
DLCO, % predicted	39.5±15.6
sPAP (excluding CVP), mmHg	43.9±12.8
LVEF, %	59.5±5.8
TAPSE, mm	21.2±2.7
pH	7.42±0.03
pO2, kPa	8.53±1.78
pCO2, kPa	5.66±1.15
Bicarbonate	26.6±3.5
mPAP at rest, mmHg	31.3±7.3
PCWP at rest, mmHg	12.8±5.6
CI at rest, L min^−1^ m^2^	3.11±0.55
PVR at rest, dyn s cm^−5^	266.5±123.5
mPAP exerc., mmHg	51.8±8.6
PCWP exerc., mmHg	23.6±9.1
CI exerc., L min^−1^ m^2^	5.3±1.3
PVR exerc., dyn s cm^−5^	245.9±72.6

BMI denotes body mass index, CI cardiac index, DLCOcarbon monoxide diffusing capacity, FEV_1_ forced expiratory volume in one second, FVC forced vital capacity, LVEF left ventricular ejection fraction, MMRC medical research council dyspnea scale, mPAP mean pulmonary artery pressure, pCO_2_ partial pressure of carbon dioxide after exercise, PCWP pulmonary capillary wedge pressure, pO_2_ partial pressure of oxygen, PVR pulmonary vascular resistance, RV residual volume, spa systolic pulmonary artery pressure (estimated), TAPSE tricuspid annular plane systolic excursion, TLC total lung capacity.

**Table 2:** Comorbidities of 16 patients with COPD and PH.

Comorbidities	
Arterial Hypertension	11 (69%)
Coronary artery disease	4 (25%)
Renal comorbidity	4 (25%)
Hypertensive heart disease	3 (19%)
Peripheral artery disease	3 (19%)
Alcohol abuse	3 (19%)
Current smoker	1 (6%)
Diabetes mellitus	1 (6%)
Malignancy	1 (6%)
Hepatic comorbidity	1 (6%)
Osteoporosis	1 (6%)

One patient fulfilling all inclusion criteria and included in the study withdrew informed consent before the first study visit. Two further patients withdrew informed consent after the first study visit. One of them experienced severe dyspnea after inhalation of the first study medication and denied to undergo any further study procedure. Retrospectively, the episode leading to study interruption followed placebo inhalation. Comprehensive mobile cardiopulmonary exercise testing, including the measures of a six-minute walk test (6MWT), was performed in 13 patients. Three patients were evaluated by standard 6MWTs. Four patients refused repeated arterial punctures and two arterial punctures were not achieved in a timely manner (21% and 4% of all arterial punctures, accordingly). Side-effects included exhaustion, dyspnea, leg weakness and headache (n = 1), and dizziness (n = 1) after low dose iloprost. Unspecific weakness was reported following the low dose iloprost (n = 2) and placebo (n = 2) inhalation.

**Figure 2:**
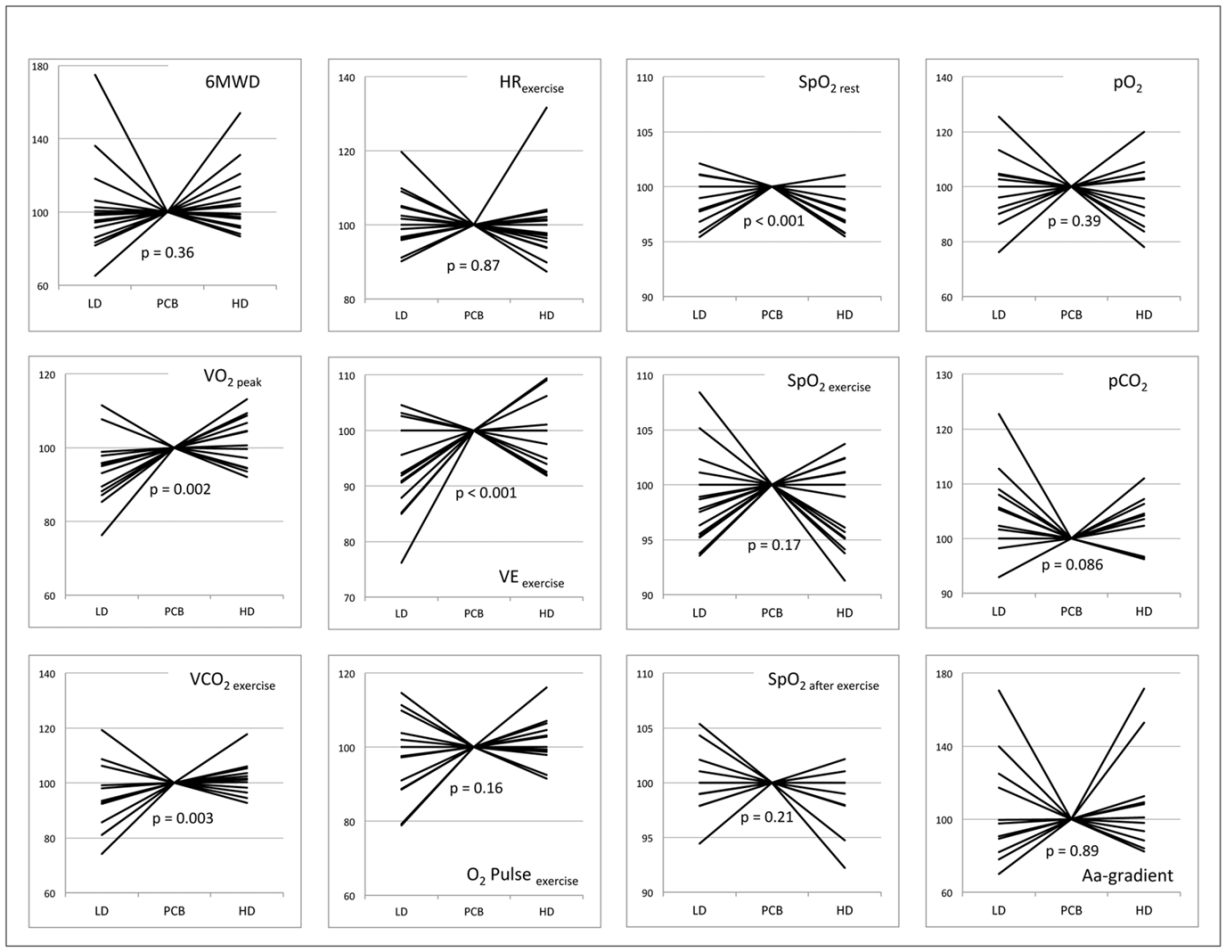
Improvement/worsening (in %) of outcome parameters in individual subjects after low dose iloprost inhalation (LD), high dose iloprost inhalation (HD) and placebo (PCB). Parameters after placebo inhalation were considered 100%. Aa-gradient denotes alveolar-arterial oxygen gradient, HR heart rate, pCO_2_ partial pressure of carbon dioxide after exercise, pO_2_ partial pressure of oxygen, SpO_2_ oxygen saturation, VCO_2_ carbon dioxide production, VE minute ventilation, VO_2_ oxygen consumption, 6MWD six-minute walking distance.

Iloprost inhalation did not affect 6-minute walking distance (p = 0.36; **Table 3**; **Figure 2**). There was a modest trend to a lower walking distance after low dose iloprost inhalation (estimated difference of the means as compared to placebo (EDOM): −12.4 m, 95% confidence interval (95% CI): −32.7–7.9 m, p = 0.22). High dose iloprost inhalation was similar to placebo in regard to walking distance (p = 0.98). Peak oxygen consumption (VO_2 peak_) during the 6MWT differed significantly over the three study groups (p = 0.002). VO_2 peak_ was clearly reduced after low dose iloprost inhalation as compared to placebo (EDOM: −76 ml/min, 95% CI: −122–−31 ml/min, p = 0.002). Noteworthy, inhalation of high dose iloprost seemed not to influence VO_2 peak_ (p = 0.92). A similar effect on carbon dioxide production during exercise (VCO_2 exercise_) was observed. Low dose iloprost impaired VCO_2 exercise_ (EDOM: −70 ml/min, 95% CI: −115–−26 ml/min, p = 0.004) whereas high dose iloprost did not alter VCO_2 exercise_ (p = 0.90). Oxygen saturation after iloprost inhalation at rest (SpO_2 rest_) was significantly different across the study groups (p<0.001). Low dose iloprost (EDOM: −1.0%, 95% CI: −1.9–−0.1%, p = 0.035) as well as high dose iloprost (EDOM: −2.2%, 95% CI: −3.1 −1.2%, p<0.001) significantly diminished SpO_2 rest_. Minimal oxygen saturation (SpO_2 exercise_) during the 6MWT was similar after placebo and iloprost inhalation (p = 0.17). However, there was a non-significant trend to a lower SpO_2 exercise_ after high dose iloprost inhalation (EDOM: −1.7%, 95% CI: −3.4–0.1%; p = 0.064). Oxygen saturation after exercise was similar in patients with low dose iloprost (p = 0.55) and declined in patients with high dose iloprost (EDOM: −2.4%, 95% CI: −3.4–0.0%; p = 0.047), as compared to placebo. Neither the partial pressure of oxygen nor the alveolar-arterial oxygen gradient was affected by treatment (p = 0.39 and 0.89, accordingly). The partial pressure of carbon dioxide after exercise was elevated in the low dose iloprost group (EDOM: 0.27 kPa, 95% CI: 0.01–0.52 kPa; p = 0.040). Ventilation during exercise (VE_ exercise_) differed in the three study groups (p<0.001). Low dose iloprost inhalation significantly impaired VE_ exercise_ (EDOM: −3.0l/min, 95% CI: −4.5–−1.5l/min, p<0.001), whereas high dose iloprost did not influence ventilation (p = 0.4). O_2_ pulse during the 6MWT was similar after iloprost and placebo inhalation (p = 0.16). Perceived exertion (BORG-scale) after the 6MWT did not differ according to the intervention (p = 0.74).

**Table 3:** Outcome parameters after inhalation of placebo, low and high dose iloprost; ^*^ denotes significant changes as compared to placebo (p<0.05); rest: after inhalation at rest before exercise, exercise: during exercise, peak: peak during exercise, after exercise: at rest after exercise; Aa-gradient denotes alveolar-arterial oxygen gradient, bpm beats per minute, n number of patients providing all three measures, pCO_2_ partial pressure of carbon dioxide after exercise, pO_2_ partial pressure of oxygen, SD standard deviation, SpO_2_ oxygen saturation, VE minute ventilation, VCO_2_ carbon dioxide production, VO_2_ oxygen consumption, 6MWD six-minute walking distance.

	n	Placebo (mean ± SD)	Iloprost 10 µg (mean ± SD)	Iloprost 20 µg (mean ± SD)
6 MWD, m	16	299±150	293±143	301±135
VO_2 rest_, ml/min	13	301±117	288±73	317±107
VO_2 peak_, ml/min	13	1018±351	946±301^*^	1024±333
VO_2 peak_, ml/min/kg	13	14.3±4.7	13.5±3.9^*^	14.3±4.4
VO_2 peak_, % pred.	13	63.9±20.2	60.5±20.9^*^	64.7±21.2
VCO_2 rest_, ml/min	13	245±113	241±79	255±88
VCO_2 exercise_, ml/min	13	903±393	842±359^*^	912±391
SpO_2 rest_, %	16	93.3±3.7	92.4±4.3^*^	91.4±3.7^*^
SpO_2 exercise_, %	16	84.1±4.8	83.0±5.0	82.6±6.0
SpO_2 after exercise_, %	16	95.1±2.7	95.0±3.5	94.1±3.9^*^
Heart rate_ rest_, bpm	16	82±16	84±15	84±17
Heart rate_ exercise_, bpm	16	109±18	110±21	109±22
Heart rate_ after exercise_, bpm	16	86±15	87±13	87±15
VE_ rest_, l/min	13	11.4±2.8	11.9±3.2	12.2±4.1
VE_ exercise_, l/min	13	35.9±18.0	33.3±17.3^*^	35.5±18.3
VE_ after exercise_, l/min	13	16.0±3.0	15.8±3.7	14.2±4.4
VR _exercise_, %	13	16.1±11.4	24.0±12.8^*^	19.1±11.5
O_2_ Pulse _exercise_, ml	13	9.4±3.0	9.1±2.6	9.5±2.9
O_2_ Pulse_ exercise_, % pred.	13	95.8±28.3	92.7±27.6	96.7±30.0
VE/VO_2 exercise_	13	33.1±7.8	32.2±7.5	33.0±9.0
VE/VCO_2 exercise_	13	38.5±8.5	37.5±6.9	38.0±8.2
pO_2_, kPa	10	7.84±2.20	7.68±1.75	6.98±1.32
pCO_2_, kPa	10	5.86±1.39	5.88±1.01^*^	6.13±1.47
Aa-gradient, kPa	10	4.28±2.17	4.51±1.68	4.82±1.51
BORG-scale	16	4.0±1.6	4.0±1.7	4.3±1.9

## Discussion

Herein we report three main findings. First, iloprost inhalation failed to improve six minute walking distance in COPD-associated PH. Second, peak oxygen consumption was not affected or declined after iloprost inhalation. Third, oxygenation at rest deteriorated following both iloprost inhalations.

The present study is the first randomized, placebo-controlled trial investigating aerosolized iloprost in patients with COPD-related PH. Against our hypothesis, inhaled iloprost did not improve exercise capacity. Low dose iloprost even impaired peak oxygen consumption during the 6MWT. However, impaired oxygen uptake did not translate into a reduced walking distance.

Multiple factors cause exercise intolerance in COPD. Ventilatory components (expiratory flow limitation, dynamic hyperinflation, respiratory muscle dysfunction), gas exchange (hypoxemia, hypercapnia) as well as peripheral factors (locomotor muscle dysfunction, deconditioning) increase energy demands and decrease energy supplies, consequently leading to dyspnea and exercise limitation [28]. In COPD-associated PH hemodynamics and cardiac output are further elements of poor exercise tolerance. Reasons for the absent exercise improvement are that the key limiting factor was not addressed, the key limiting factor was insufficiently addressed or a simultaneous deterioration of another exercise limiting component.

A small number of other vasodilators were investigated in COPD so far. Nifedipine caused de-oxygenation in patients with COPD, most likely due to inhibition of hypoxic vasoconstriction [29], [30]. Bosentan, an endothelin-receptor antagonist worsened oxygenation and quality of life in patients with severe COPD [10]. Moreover, three months of sildenafil did not improve exercise capacity in a similar cohort of COPD patients [11]. In a recent study, sildenafil deteriorated oxygenation due to impaired V/Q distributions at rest [31]. Still, there was a trend to a higher V/Q imbalance during exercise. Nevertheless, a beneficial effect of pulmonary vasodilators on hemodynamics is also in COPD-related PH likely [31], [32]. Probably, most drugs lack selective vasodilative properties and consequently inhibit hypoxic vasoconstriction. Iloprost, a rather large molecule with a short half-life, administered via inhalation, is one of the most selective pulmonary vasodilators available yet. However, similar flaws of inhaled iloprost limit its utilization in COPD.

Iloprost inhalation clearly impaired oxygenation at rest, both at low and high dose. An enhanced perfusion, similar to the effect reached by other pulmonary vasodilators, most likely caused a worsening of V/Q mismatch. It is unclear, whether selectivity of inhaled iloprost is insufficient or vasodilation *per se* causes impaired V/Q matching in COPD patients at rest. Although not statistically significant, there was a dose dependent decrease in oxygen saturation during exercise. Six minutes after exercise, once more at rest, oxygen saturation was clearly diminished after high dose iloprost inhalation. Thus, it is likely that the effect of iloprost on oxygenation is more pronounced at rest. A similar phenomenon was observed after sildenafil administration [31]. Noteworthy, beneficial effects of sildenafil on hemodynamics were similar at rest and during exercise.

Besides V/Q mismatch, other mechanisms impairing oxygenation and exercise capacity are conceivable. The major factor limiting exercise capacity in most COPD patients is ventilation. A minor effect of iloprost on airways could potentially aggravate air trapping, dynamic hyperinflation and compromise exercise tolerance. In accordance, decreased minute ventilation was observed after low dose iloprost inhalation. Our results are in line with results of a previous study, which suggest a significantly lower minute ventilation after iloprost inhalation [20]. The elevated carbon dioxide levels following iloprost inhalation might either reflect an effect on the ventilatory pump or an impairment of the respiratory drive. Previously, it was reported that iloprost attenuates cerebral blood flow, which might alter ventilatory control [33].

In the current trial, the subgroup of COPD patients who improved in walking distance also improved in peak oxygen consumption. These three patients were characterized by a more restricted diffusion capacity, more dyspnea and a more severe right cardiac strain, while left cardiac function was preserved (data not shown). Thus, patients with a more severe hemodynamic and right cardiac compromise might still benefit. Presumably, ventilatory factors were the main determinants of exercise intolerance in this population. This is also in line with a recent study, suggesting that only COPD patients with severe PH (mPAP≥40 mmHg) are limited by hemodynamics [34]. Moreover, the deleterious effects of iloprost on gas exchange and potentially on ventilation have further precluded an exercise improvement.

A few limitations of the study need to be mentioned. Obtained results are restricted to acute effects of the investigated iloprost dose. Whether other or repeated medication doses would have yielded another outcome is not known. However, our results already prove a clinically relevant deterioration of oxygenation at rest following single iloprost inhalation. Thus, a) indicating that there is a measurable (detrimental) effect of inhaled iloprost in this population; and b) questioning the appropriateness of a long-term trial in respect of short-term safety. The fact that all patients receiving continuous open-label iloprost inhalation following the study end, discontinued therapy within four weeks due to side-effects and/or lack of subjective clinical benefit, supports this notion. Despite careful patient evaluation we cannot rule out that other causes of PH contributed to elevated pulmonary pressures. A considerable number of patients had cardiac comorbidities and six patients had slightly elevated pulmonary capillary wedge pressures (PCWP≥15 mmHg). Since left ventricular pressures were not measured it remains uncertain whether this reflects a true elevation [35]. Importantly, all patients had a preserved systolic function. However, a minor cardiac dysfunction at rest might have become more relevant during exercise. Three patients did not achieve current criteria for pulmonary hypertension (mPAP>25 mmHg). Mean pulmonary pressures during exercise in these patients were 49, 58 and 62 mmHg, respectively. Due to a significant number of missing arterial blood gases (25%) interpretation is limited.

In summary, inhaled iloprost failed to improve exercise capacity in patients with COPD-related PH. Negative effects of inhaled iloprost on oxygenation limit applicability in COPD. Up to date inhaled iloprost cannot be recommended in patients with COPD.

## Supporting Information

Checklist S1 **CONSORT Checklist.**
(DOC)

Protocol S1 **Trial Protocol.**
(DOC)

Supporting Information S1 **A detailed description of the methods.**
(DOC)
