# Supplementary material for: Acute Effects of Aerosolized Iloprost in COPD Related Pulmonary Hypertension - A Randomized Controlled Crossover Trial
Source: PLoS One. 2012 Dec 27;7(12):e52248. doi: 10.1371/journal.pone.0052248 (PMC3531427; doi:10.1371/journal.pone.0052248)
Supplement: Protocol S1 — Trial Protocol. (DOC) [file pone.0052248.s002.doc]

**OPTION – On demand ProsTacyclin Inhalation in Obstructive pulmonary disease aNd pulmonary hypertension**

Lucas Boeck, Anja Meyer, Michael Tamm, Daiana Stolz

Pneumology, University Hospital Basel, Switzerland

# PROTOCOL SYNOPSIS

| **Study title** | **OPTION – On demand ProsTacyclin Inhalation in Obstructive pulmonary disease aNd pulmonary hypertension** |
| --- | --- |
| **Background** | Secondary pulmonary hypertension (PH) is a common complication of chronic obstructive pulmonary disease (COPD), worsening the clinical course of disease. Various pathophysiological mechanisms contribute to the development of PH at rest and during exercise. However, there is a lack of general understanding and differences to primary pulmonary arterial hypertension (PAH) remain unclear. Worsening of PH during exercise stresses cardiac function and might be the main factor of exercise limitation. Vasodilatatory agents such as calclium-channel blocker, nitric oxide, phosphodiesterase type 5 inhibitors and endothelin-receptor antagonists used in PAH seem not to be benefitial in COPD. Prostanoids, with a vasodilatatory and antiproliferative profile, have not been investigated so far in COPD, where a decreased production of prostacyclin in endothelial cells was shown. Inhalative iloprost, providing a very good risk profile, might be an effective drug improving exercise tolerance in secondary PH due to COPD. Furthermore an inhalative application facilitates the access to those alveolar units which receive the greatest proportion of ventilation, therefore potentially minimizing V/Q mismatch while improving cardiac adaptability during exercise. |
| **Objectives** | Evaluation of the short time effectiveness and safety of the inhaled prostacyclin iloprost in COPD patients with secondary pulmonary hypertension. |
| **Study design** | This in an investigator driven single center prospective randomized double-blind study including a total of 17 patients.  1^st^ study visit: informed consent, clinical history, physical examination, quality of life questionnaires (SF-36/SGRQ), pre- and postbronchodilator bodyplethysmography, arterial blood gas analyses, patients will be blinded assigned to inhaled iloprost (either 10µg or 20µg) or inhaled placebo (NaCl 0.9%), mobile spiroergometry after inhalation, second blood gas analysis after exercise  2^nd^ and 3^rd^ visit: randomization to substance/dose, mobile spiroergometry, arterial blood gas analyses after exercise |
| **Population** | COPD patients with disproportional PH (mean pulmonary artery pressure of over 45mmHg during exercise and/or over 30mmHg at rest) |
| **Primary Endpoint** | 6 minute walking distance |
| **Secondary Endpoints** | oxygen consumption, oxygen saturation, ventilation, carbon dioxide production, arterial oxygen content, Alveolar-arterial gradient, BORG score |
| **Study medication** | Inhalative Iloprost (Ilomedin®) 10µg/20µg, diluted in saline (NaCl 0.9%), inhalation through an ultrasonic nebulizer system (Multisonic® infracontrol) |
| **Number of subjects** | 17 |
| **Principle Investigator** | PD Dr. med. Daiana Stolz, University Hospital Basel |

# TABLE OF CONTENTS

[1 PROTOCOL SYNOPSIS 2](#__RefHeading___Toc231713445)

[2 TABLE OF CONTENTS 3](#__RefHeading___Toc231713446)

[3 INTRODUCTION 4](#__RefHeading___Toc231713447)

[4 STUDY OBJECTIVES 7](#__RefHeading___Toc231713448)

[5 METHODS 8](#__RefHeading___Toc231713449)

[5.1 Study population 8](#__RefHeading___Toc231713450)

[5.1.1 Inclusion criteria 8](#__RefHeading___Toc231713451)

[5.1.2 Exclusion criteria 8](#__RefHeading___Toc231713452)

[5.2 Study design 9](#__RefHeading___Toc231713453)

[6 STATISTICAL ANALYSES 11](#__RefHeading___Toc231713454)

[7 ADVERSE EVENTS 12](#__RefHeading___Toc231713455)

[8 TIME SCHEDULE 13](#__RefHeading___Toc231713456)

[9 INFORMED CONSENT, ETHICAL REVIEW, LIABILITY AND REGULATORY CONSIDERATIONS 14](#__RefHeading___Toc231713457)

[10 PROTOCOL SIGNATURE 15](#__RefHeading___Toc231713458)

[11 REFERENCES 16](#__RefHeading___Toc231713459)

[12 ATTACHMENTS 18](#__RefHeading___Toc231713460)

[12.1 SF-36 Questionaire 18](#__RefHeading___Toc231713461)

[12.2 SGR Questionaire 19](#__RefHeading___Toc231713462)

[12.3 BORG score 20](#__RefHeading___Toc231713463)

# INTRODUCTION

Secondary pulmonary hypertension (PH) is a common complication of chronic obstructive pulmonary disease (COPD). Due to a lack of systematical investigations the actual incidence of PH in COPD is unclear. However, different studies assessed an elevated pulmonal artery pressure (PAP) from 20% up to 91% of the COPD patients [1, 2]. Similar to other causes, PH has a tremendous impact on outcome in COPD. Several studies before and after the introduction of long term oxygen therapy showed that an increasing pulmonary artery pressure was associated with a progressive decline in survival [3, 4]. It was also suggested as the single best predictor of mortality in COPD [5], as well as a predictor for severe exacerbations [6]. In patients with moderate COPD and normal PAP at rest, 58% developed pulmonary hypertension during exercise. This subpopulation was at increased risk for developing PH at rest [7]. Usually progression of PH in COPD is slow and PAP is only moderately elevated, even in patients with advanced disease [2]. However, there is a minority of patients with mainly moderate airflow obstruction, who develop severe PH. In this subgroup with “dysproportional hypertension” survival and symptoms are rather driven by the pulmonary vascular component then by airflow.

The main principles in developing PH in COPD are hypoxic-vasoconstriction, vascular remodeling and endothelial dysfunction. In continuously decreased alveolar oxygen partial pressures, hypoxic vasoconstriction is assumed as the mechanism for developing PH and subsequent vascular remodeling. It plays an important role in matching blood flow to ventilation. However, absent in the early stages [8], not reversing PH [9], the impact of hypoxia on PAP in COPD is questionable. The inhibition of this mechanism, among others with oxygen, has shown a worsening in ventilation/perfusion (V/Q) distribution [10], limiting therapeutical options. Worsening is lower in patients with severe structural impairment of the pulmonary arteries [8]. Furthermore the magnitude of constriction is altered by the endothelial function [11]. Remodeling of pulmonary vessels consists of intimal changes, showing an increase in smooth muscle cells and fibrosis [12], and of muscularisation, regarding arterioles, postcapillary vessels and larger pulmonary arteries [13]. Endothelial dysfunction in pulmonary arteries exists from mild disease on [14]. Mediators such as prostacyclin, nitric oxide, endothelin-1 and angiotensin are responsible for balanced vascular actions [10]. As other important mechanisms inflammation, hypercapnic acidosis and polycythaemia are considered [15].

During exercise, PAP is increasing, especially in patients with preexisting PH at rest [1]. Additionally to the mechanisms discussed above, reduction of the capillary bed in emphysema and the compression by increased alveolar pressure, due to exspiratory flow limitation, are involved. Exclusive PH during exercise may exist years before an elevated pressure emerges at rest [7]. In very severe COPD, massive swings in intrathoracic pressure, resulting from deranged ventilatory mechanics are the most likely cause of exercise limitation [16]. The contributions of different factors such as hypoxia, deconditioning and heart disease remain unknown. But studies have shown that the stroke volume may be reduced in COPD and frequently fails to raise during exercise, resulting in an decreased oxygen delivery and possible impaired exercise capacity [17].

Oxygen is the only substance with proven mortality benefit in treating PH in COPD. Vasodilatating agents improve symptoms, exercise tolerance and survival in primary pulmonary hypertension. Despite different pathophysiological causes in COPD, it was assumed that by reduction of PAP, improving right cardiac function and oxygen delivery, vasodilators might increase exercise tolerance and eventually survival [10]. But PH in COPD appears to be different. Nifedipine, a calcium-channel blocker, reduced PAP and increased cardiac output in COPD patients. However, inhibition of hypoxic vasoconstriction induced worsening of V/Q mismatch resulting in lower arterial oxygen content [18, 19]. Nitric oxide inhalation worsened gas exchange at rest, due to increasing V/Q mismatch. A slight benefit of inhalative nitric oxide was shown during exercise. It moderately reduced PAP, without worsening V/Q relation and may prevent exercise induced oxygen desaturation [20]. Furthermore bosentan, an endothelin-receptor antagonist, has been shown to cause oxygen desaturation, reduction of quality of life and missed the primary endpoint of improving the 6-minute walking distance in severe COPD [21]. Sildenafil, a phosphodiesterase type 5 inhibitor, was not able to improve exercise capacity in COPD patients [22].

So far, the only class of vasodilators not investigated in COPD are prostanoids. Prostacyclin (Prostaglandin I_2_) produced by the vascular endothelium is a potent vasodilatator, inhibits muscular proliferation and platelet aggregation. In patients with primary pulmonary hypertension, PH associated with connective-tissue disease and thrombembolic process, inhalative iloprost could achieve an increase in the 6-minute walking distance and improve NYHA functional class [23]. In COPD, dysfunctional pulmonary-artery endothelial cells have a decreased production of prostacyclin. Therefore, an imbalance in eicosanoid expression may be central in COPD related endothelial dysfunction. Furthermore, prostacyclin has protective effects in the pulmonary vasculature after cigarette exposure [24]. In two case reports, epoprostenol and inhalative iloprost, a synthetic prostacyclin analogue, have shown a benefit in severe PH in COPD [25, 26]. Inhalative treatment facilitates the access of iloprost to those alveolar units which receive the greatest proportion of ventilation, hence minimizing V/Q mismatch while improving cardiac adaptability during exercise.

To summarize the latest findings: no vasodilator has shown to be beneficial in COPD/ or COPD related PH, inhalative treatment seems to be favorably against systemical therapy, there is evidence that short acting vasodilators might improve exercise capacity, prostacyclin/prostacyclin derivates have not been investigated in COPD

Taken the drawbacks of the past, inhalative iloprost, providing a very good risk profile, could be a promising drug in treating PH in COPD, which needs to be evaluated. Therefore we hypothesize that inhaled iloprost on demand (before exercise) improves exercise capacity and is well tolerated in patients with COPD and PH during exercise.

# STUDY OBJECTIVES

The primary objective of this trial is to evaluate the short time effectiveness and safety of the inhaled prostacyclin iloprost in COPD patients with secondary pulmonary hypertension.

Primary endpoint:

- Improvement in 6 minutes walking distance immediately after iloprost inhalation as compared to placebo

Secondary endpoints:

- Oxygen consumption (VO_2_ max), oxygen saturation (SaO_2_), Ventilation (VE), carbon dioxide production (VCO_2_) as measured by the mobile exercise test
- Changes in pO_2_, Alveolar-arterial gradient measured by arterial blood gas analyses
- Changes in BORG score

# METHODS

## Study population

The trial is designed as a double-blinded crossover study carried out at the University Hospital Basel, Switzerland.

A total of 17 patients will be recruited. Patients or their legal representatives are required to provide written informed consent that they agree to participate in the study.

### Inclusion criteria

- Patients with diagnosed COPD (chronic non-reversible obstruction with a FEV_1_/FVC under 0.7)
- Confirmed disproportional pulmonary arterial hypertension (mean pulmonary artery pressure of over 45mmHg during exercise and/or over 30mmHg at rest, measured at any time before inclusion).

### Exclusion criteria

- mental disorder preventing appropriate judgment concerning study participation
- significant comorbidity resulting in reduced life expectancy (lower than 6 months)
- significant exacerbation of COPD within the last month
- decompensated heart failure (left ventricular ejection fraction below 30%)
- present pulmonary embolism
- PH explained by another cause than COPD (e.g. collagen vascular diseases, congenital shunts, HIV, use of anorexigens, interstitial lung disease, sarcoidosis, histiocytosis X)
- pregnant and breastfeeding women

## Study design

Patients with pulmonary hypertension attending the pulmonary clinical department at the University Hospital of Basel, Switzerland and meeting the study inclusion criteria will be considered for eligibility. Before starting, patients should be on optimal inhalative therapy for at least two weeks (triple therapy of long-acting beta2-agonist, long-acting anticholinergics and inhaled corticosteroids). Oxygen therapy should be administered according to the guidelines. In patients with insufficient therapy, missing drugs will be started and inclusion postponed. Women in childbearing age, not taking reliable contraception, will undergo a pregnancy testing.

Overall three study visits are planned. In the first visit, after providing informed consent, patients will undergo clinical history taking and physical examination. Patients will complete quality of life questionnaires (SF-36/SGRQ). Pre and postbronchodilator bodyplethysmography will be performed. Arterial blood for an ABGA will be sampled. Thereafter, patients will be blinded assigned to one of the two treatments: inhaled iloprost (either 10µg or 20µg) or inhaled placebo (NaCl 0.9%). Randomization will be computer-based. An additional study assistant, who is not involved in the rest of the trial, will randomize each patient computer-based and prepare inhalation solutions. All other involved (blinded) persons will receive this information at the end of the study.

Patients randomized to iloprost will inhale iloprost diluted in saline (NaCl 0.9%) through an ultrasonic nebulizer system [27] (Multisonic® infracontrol; Schill, Probstzella, Germany). A total dose of 10µg/20µg Iloprost (Ilomedin®) will be administered in a 2ml solution. Mobile spiroergometry will be carried out shortly after. A second blood gas analysis will be performed after spiroergometry. In the second and third visit, the patients will be again randomized to the substance/dose. Mobile spiroergometry and arterial blood gas analysis will be performed in the same manner.

Table: Diagram of the examinations/interventions planned during the study

|  | **visit 1**  week 1 | **visit 2**  week 2 | **visit 3**  week 3 |
| --- | --- | --- | --- |
| Informed consent | x |  |  |
| History | x |  |  |
| Physical examination | x |  |  |
| Vitals | x | x | x |
| Spirometry pre/post BD | x |  |  |
| SF-36/ SGRQ | x |  |  |
| Blinded inhalation of   - Placebo - Iloprost 10µg - Iloprost 20µg | x | x | x |
| Mobile spiroergometry | x | x | x |
| Arterial blood gas analyses | x | x | x |
| BORG-score | x | x | x |

# STATISTICAL ANALYSES

Power was calculated using the 6 minutes walk test distance before and after treatment as the primary outcome variable. Assuming a standard deviation of the difference before and after treatment of 50m, there is a power >85% to detect a mean difference of 40m with a sample size of 17 subjects by a two sided paired T-Test (alpha=0.05).

Analysis of the primary end-point will be evaluated on the intention-to-treat principle. To detect an improvement in the 6 minutes walk test before and after treatment a paired T-test will be performed. A 95% confidence interval of the mean of differences will also be calculated. Descriptive statistics as mean, SD, median, min, max will be calculated for all variables. Secondary variables will be analyzed using paired T-tests for approximate normal distributed variables, Wilcoxon-tests for ordinal variables and Fisher’s exact tests for nominal variables as appropriate. Statistical analyses will be performed by using SPSS (Statistical Package for Social Sciences, version 16.0 for Windows; Chicago, IL) and R.

# ADVERSE EVENTS

All clinical events occurring during the study period will be monitored. Serious and unexpected adverse events leading to death or permanent disability will be reported to the ethical committee within 24 hours. The possible causality between Iloprost and adverse events will be evaluated.

# TIME SCHEDULE

Submission to the ethical committee: July 2009

Patient enrollment & follow-up: August 2009 to November 2010

Data Analysis: November-December 2010

# INFORMED CONSENT, ETHICAL REVIEW, LIABILITY AND REGULATORY CONSIDERATIONS

**7.1 Informed consent**

Patients are required to provide written informed consent, that they agree to participate in the study, before enrollment. The document explains in simple terms the aim of the study, required visits and procedures. It contains a statement that the consent is freely given, and that the patient is free to withdraw from the study at any time.

**7.2 Ethical review**

This protocol will be submitted for approval to the conjoined local ethic committee of the cantons of Basel-Stadt and Basel-Land.

**7.3 Liability**

After having been approved by the ethical committee, the diagnostic procedures will be covered by the general liability insurance RIMAS* of the University Hospital Basel, as certified by Jürg Müller.

**7.4 Regulatory Considerations**

This study will be conducted in accordance with the ethical principles stated in the most recent version of the Declaration of Helsinki or the applicable International Conference on Harmonization (ICH) guidelines on good clinical practice, whichever represents the greater protection of the individual.

# PROTOCOL SIGNATURE

I have read the protocol and agree to conduct the study as outlined.

____________________________________

Prof. Dr. med. Michael Tamm

____________________________________

PD Dr. med Daiana Stolz

____________________________________

Dr. med. Lucas Böck

# REFERENCES

1. Burrows B, Kettel LJ, Niden AH, Rabinowitz M, Diener CF. Patterns of cardiovascular dysfunction in chronic obstructive lung disease. *N Engl J Med* 1972: 286(17): 912-918.

2. Scharf SM, Iqbal M, Keller C, Criner G, Lee S, Fessler HE. Hemodynamic characterization of patients with severe emphysema. *Am J Respir Crit Care Med* 2002: 166(3): 314-322.

3. Bishop JM. Role of hypoxia in the pulmonary hypertension of chronic bronchitis and emphysema. *Scand J Respir Dis Suppl* 1971: 77: 61-65.

4. Dallari R, Barozzi G, Pinelli G, Merighi V, Grandi P, Manzotti M, Tartoni PL. Predictors of survival in subjects with chronic obstructive pulmonary disease treated with long-term oxygen therapy. *Respiration* 1994: 61(1): 8-13.

5. Oswald-Mammosser M, Weitzenblum E, Quoix E, Moser G, Chaouat A, Charpentier C, Kessler R. Prognostic factors in COPD patients receiving long-term oxygen therapy. Importance of pulmonary artery pressure. *Chest* 1995: 107(5): 1193-1198.

6. Kessler R, Faller M, Fourgaut G, Mennecier B, Weitzenblum E. Predictive factors of hospitalization for acute exacerbation in a series of 64 patients with chronic obstructive pulmonary disease. *Am J Respir Crit Care Med* 1999: 159(1): 158-164.

7. Kessler R, Faller M, Weitzenblum E, Chaouat A, Aykut A, Ducolone A, Ehrhart M, Oswald-Mammosser M. "Natural history" of pulmonary hypertension in a series of 131 patients with chronic obstructive lung disease. *Am J Respir Crit Care Med* 2001: 164(2): 219-224.

8. Barbera JA, Riverola A, Roca J, Ramirez J, Wagner PD, Ros D, Wiggs BR, Rodriguez-Roisin R. Pulmonary vascular abnormalities and ventilation-perfusion relationships in mild chronic obstructive pulmonary disease. *Am J Respir Crit Care Med* 1994: 149(2 Pt 1): 423-429.

9. Weitzenblum E, Schrijen F, Mohan-Kumar T, Colas des Francs V, Lockhart A. Variability of the pulmonary vascular response to acute hypoxia in chronic bronchitis. *Chest* 1988: 94(4): 772-778.

10. Barbera JA, Peinado VI, Santos S. Pulmonary hypertension in chronic obstructive pulmonary disease. *Eur Respir J* 2003: 21(5): 892-905.

11. Peinado VI, Santos S, Ramirez J, Roca J, Rodriguez-Roisin R, Barbera JA. Response to hypoxia of pulmonary arteries in chronic obstructive pulmonary disease: an in vitro study. *Eur Respir J* 2002: 20(2): 332-338.

12. Wright JL, Petty T, Thurlbeck WM. Analysis of the structure of the muscular pulmonary arteries in patients with pulmonary hypertension and COPD: National Institutes of Health nocturnal oxygen therapy trial. *Lung* 1992: 170(2): 109-124.

13. Magee F, Wright JL, Wiggs BR, Pare PD, Hogg JC. Pulmonary vascular structure and function in chronic obstructive pulmonary disease. *Thorax* 1988: 43(3): 183-189.

14. Peinado VI, Barbera JA, Ramirez J, Gomez FP, Roca J, Jover L, Gimferrer JM, Rodriguez-Roisin R. Endothelial dysfunction in pulmonary arteries of patients with mild COPD. *Am J Physiol* 1998: 274(6 Pt 1): L908-913.

15. Chaouat A, Naeije R, Weitzenblum E. Pulmonary hypertension in COPD. *Eur Respir J* 2008: 32(5): 1371-1385.

16. Montes de Oca M, Rassulo J, Celli BR. Respiratory muscle and cardiopulmonary function during exercise in very severe COPD. *Am J Respir Crit Care Med* 1996: 154(5): 1284-1289.

17. Bogaard HJ, Dekker BM, Arntzen BW, Woltjer HH, van Keimpema AR, Postmus PE, de Vries PM. The haemodynamic response to exercise in chronic obstructive pulmonary disease: assessment by impedance cardiography. *Eur Respir J* 1998: 12(2): 374-379.

18. Melot C, Hallemans R, Naeije R, Mols P, Lejeune P. Deleterious effect of nifedipine on pulmonary gas exchange in chronic obstructive pulmonary disease. *Am Rev Respir Dis* 1984: 130(4): 612-616.

19. Agusti AG, Barbera JA, Roca J, Wagner PD, Guitart R, Rodriguez-Roisin R. Hypoxic pulmonary vasoconstriction and gas exchange during exercise in chronic obstructive pulmonary disease. *Chest* 1990: 97(2): 268-275.

20. Roger N, Barbera JA, Roca J, Rovira I, Gomez FP, Rodriguez-Roisin R. Nitric oxide inhalation during exercise in chronic obstructive pulmonary disease. *Am J Respir Crit Care Med* 1997: 156(3 Pt 1): 800-806.

21. Stolz D, Rasch H, Linka A, Di Valentino M, Meyer A, Brutsche M, Tamm M. A randomised, controlled trial of bosentan in severe COPD. *Eur Respir J* 2008: 32(3): 619-628.

22. Rietema H, Holverda S, Bogaard HJ, Marcus JT, Smit HJ, Westerhof N, Postmus PE, Boonstra A, Vonk-Noordegraaf A. Sildenafil treatment in COPD does not affect stroke volume or exercise capacity. *Eur Respir J* 2008: 31(4): 759-764.

23. Olschewski H, Simonneau G, Galie N, Higenbottam T, Naeije R, Rubin LJ, Nikkho S, Speich R, Hoeper MM, Behr J, Winkler J, Sitbon O, Popov W, Ghofrani HA, Manes A, Kiely DG, Ewert R, Meyer A, Corris PA, Delcroix M, Gomez-Sanchez M, Siedentop H, Seeger W. Inhaled iloprost for severe pulmonary hypertension. *N Engl J Med* 2002: 347(5): 322-329.

24. Nana-Sinkam SP, Lee JD, Sotto-Santiago S, Stearman RS, Keith RL, Choudhury Q, Cool C, Parr J, Moore MD, Bull TM, Voelkel NF, Geraci MW. Prostacyclin prevents pulmonary endothelial cell apoptosis induced by cigarette smoke. *Am J Respir Crit Care Med* 2007: 175(7): 676-685.

25. Hegewald MJ, Elliott CG. Sustained improvement with iloprost in a COPD patient with severe pulmonary hypertension. *Chest* 2009: 135(2): 536-537.

26. Ishikawa S, Yano S, Wakabayashi K, Tokuda Y, Kobayashi K, Ikeda T, Takeyama H. [A case of severe pulmonary hypertension associated with COPD treated with epoprostenol]. *Nihon Kokyuki Gakkai Zasshi* 2008: 46(8): 660-666.

27. Gessler T, Schmehl T, Hoeper MM, Rose F, Ghofrani HA, Olschewski H, Grimminger F, Seeger W. Ultrasonic versus jet nebulization of iloprost in severe pulmonary hypertension. *Eur Respir J* 2001: 17(1): 14-19.

# ATTACHMENTS

## SF-36 Questionaire

## SGR Questionaire

## BORG score
