# Supplementary material for: Acute Effects of Aerosolized Iloprost in COPD Related Pulmonary Hypertension - A Randomized Controlled Crossover Trial
Source: PLoS One. 2012 Dec 27;7(12):e52248. doi: 10.1371/journal.pone.0052248 (PMC3531427; doi:10.1371/journal.pone.0052248)
Supplement: Supporting Information S1 — A detailed description of the methods. (DOC) [file pone.0052248.s003.doc]

**Aerosolized iloprost in COPD-related pulmonary hypertension**

**– a randomized, placebo-controlled trial**

*Lucas Boeck, Michael Tamm, Peter Grendelmeier and Daiana Stolz*

**Supplementary material**

**METHODS**

Pulmonary Function Testing

Dynamic and static pulmonary function parameters were evaluated by body plethysmography. Carbon monoxide diffusing capacity (DLCO) was measured. All tests were performed according to the European Respiratory Society standards .

Echocardiography

Transthoracic two-dimensional and Doppler echocardiography were carried out by an ultrasound instrument (Philips, iE33) and performed in all patients for screening. Two-dimensional and Doppler imaging was performed in standard parasternal and apical views. Systolic pulmonary artery pressures (sPAP) were estimated from the systolic transtricuspid pressure gradient by means of the modified Bernoulli equation (tricuspid pressure gradient = 4 x [maximal velocity of tricuspid regurgitant jet]^2^ ). Tricuspid annular motion (TAM) was estimated by two-dimensional echo guided M-mode recordings from the apical four-chamber view.

Right Heart Catheterization

Right heart catheterization was performed in patients with elevated sPAP and / or right cardiac dysfunction. Standard measurements were taken without sedation from the right atrium, right ventricle and pulmonary artery at the end of expiration. Cardiac output was determined by the thermodilution method, whereas cardiac index is corrected for body surface area. Patients with normal or slightly elevated pulmonary pressures at rest were further evaluated during exercise.

Randomization

Formulas for inhalation were randomized by an automated computer-generated randomization scheme and assigned to specific study days. The patient as well as the study personnel, who administered the inhalation and performed all tests, was blinded to the medication allocation. A nurse and a physician, responsible for preparation of the medication, were the only persons aware of the randomization code during the trial. They were not involved in other study functions.

Inhalation

Every patient received 10μg iloprost (low dose iloprost), 20 μg iloprost (high dose iloprost) or placebo (normal saline) on three different study days. Iloprost (Ventavis®) was diluted in normal saline to achieve a 2ml solution. The three solutions were not visually distinguishable. Approximately 15 minutes before inhalation of the study medication a short acting beta-2 agonist (200μg salbutamol; Ventolin®) was inhaled via a spacer. Placebo and iloprost was inhaled through an ultrasonic nebulizer system (Multisonic® infracontrol; Schill, Probstzella, Germany) .

Mobile Cardiopulmonary Exercise Testing

The mobile cardiopulmonary exercise test was performed exactly 10 minutes after iloprost or placebo inhalation as described previously . Exercise parameters were measured using a telemetric mobile cardiopulmonary exercise test device (Oxycon Mobile® software v. 4.6, VIASYS Healthcare GmbH, Würzburg, Germany). This device consists of an EKG-triggered belt, an oxygen sensor, a facemask with a dead space < 30 ml, a flow sensor, a sensor unit to measure oxygen and carbon dioxide, a data storage unit and a data transfer unit with integrated long-range telemetry, allowing real-time monitoring of the data. Heart rate, oxygen saturation, respiratory rate, tidal volume, oxygen consumption (VO2) and carbon dioxide production (VCO2) were continuously registered. Out of those parameters ventilation (VE), ventilatory reserve, O2 pulse, and ventilatory equivalents (VE/VO2, VE/CO2) were calculated. Before the exercise test there was a resting phase of several minutes. Measures at rest were obtained during a steady state after inhalation before exercise start. 10 minutes after inhalation of iloprost / placebo a six-minute walking test (6MWD) with the mobile exercise equipment was performed according to the American Thoracic Society guidelines . At all times patients had the opportunity to slow the pace, to stop temporarily and to discontinue the test. During the test patients were not encouraged to walk faster or to continue walking. Exercise measures were obtained at peak oxygen uptake. Except for oxygen saturation, where minimal values, and heart rate where maximal values during exercise were analyzed. After test measures were acquired 6 minutes after exercise stop. Walking distance, perceived dyspnea as well as complications / side effects were recorded after the tests.

Six-Minute Walk Test

The 6MWT without cardiopulmonary exercise equipment was performed in patients who did not tolerate the facemask and / or strongly required oxygen during exercise. Inhalation, resting and the 6MWT was carried out identically. Heart rate and oxygen saturation were monitored continuously throughout the test. Walking distance, perceived dyspnea and complications / side effects were recorded after the test. 6MWTs were performed with the same amount of oxygen in each patient.

Arterial Blood Gas Analysis

Immediately after each exercise test a standard arterial puncture to obtain a specimen for blood gas analysis was performed.

Power calculation

Power was calculated using the 6MWT distance before and after treatment as the primary outcome variable. Assuming a standard deviation of the difference before and after treatment of 50m, there is a power > 80% to detect a mean difference of 40m with a sample size of 16 subjects by a two sided paired t-test (alpha = 0.05).

**Definitions**

Air trapping residual volume / total lung capacity > 0.4

Emphysema diffusion capacity of carbon monoxide < 70% predicted

Hypercapnia partial pressure of carbon dioxide > 6kPa

Hyperinflation total lung capacity > 120% predicted

Hypoxemia partial pressure of oxygen < 8kPa

Severe pulmonary hypertension mPAP at rest > 40mmHg

Severe COPD COPD GOLD 3

Very severe COPD COPD GOLD 4

**REFERENCES**

1 Quanjer PH, Tammeling GJ, Cotes JE, Pedersen OF, Peslin R, Yernault JC. Lung volumes and forced ventilatory flows. Report Working Party Standardization of Lung Function Tests, European Community for Steel and Coal. Official Statement of the European Respiratory Society. *Eur Respir J Suppl* 1993; **16:** 5-40.

2 Gessler T, Schmehl T, Hoeper MM*, et al.* Ultrasonic versus jet nebulization of iloprost in severe pulmonary hypertension. *Eur Respir J* 2001; **17:** 14-9.

3 Attinger A, Tuller C, Souren T, Tamm M, Schindler C, Brutsche MH. Feasibility of mobile cardiopulmonary exercise testing. *Swiss Med Wkly* 2006; **136:** 13-8.

4 Tueller C, Kern L, Azzola A*, et al.* Six-minute walk test enhanced by mobile telemetric cardiopulmonary monitoring. *Respiration; international review of thoracic diseases* 2010; **80:** 410-8.

5 ATS statement: guidelines for the six-minute walk test. *American journal of respiratory and critical care medicine* 2002; **166:** 111-7.
